# Health outcomes, education, healthcare delivery and quality – 3060. Assessment of parameters of six-minute walk test (6MWT) in healthy young subjects

**DOI:** 10.1186/1939-4551-6-S1-P227

**Published:** 2013-04-23

**Authors:** Prasad Eswara Chelluri, Sridhar Pulluri, Ragender Mukka, Sathyaprasad Andru

**Affiliations:** 1 Department of Pulmonolgy, Shadan Institute of Medical Sciences, Hyderabad, India; 2 Pulmonology, Mamata Medical College, Khammam, India; 3 Pulmonology, Chelmeda Ananda Rao Institute of Medical Sciences, Karimnagar, India

## Aim

Perform 6MWT in healthy young adults and its determining factors.

## Background

Functional exercise capacity can be assesed by several modalities. 6MWT provides a better tolerated, easy to adminster, daily life activity test. American Thoracic Society (ATS) provides guidelines for 6MWT (2002). This is a simple test which can be performed at primary care level and recommended by guidelines committee for COPD in India (2003). There are hardly any publications on 6MWT in normal healthy individuals of India. The present study is a sample survey of 6MWT in heathy young college students.

## Methods

A cross-sectional study was conducted in 300 healthy subjects (male-200, female-100). Age, height in cms, weight in kg baseline and post performance pulse rate were recorded. 6MWT was conducted in a 100 ft long halfway in the medical college using lap counter to count the number of laps completed and alarm that sounded at six minutes after the walk started. Distance covered at the end of the six minutes was noted as well as pulse rate. ATS guidelines were followed. Statistical analysis was performed by SPSS16 to calculate person correlation coefficient and student t-test.

## Results

Subjects: male-200, female-100. Table 1

**Table1:** 

Age	male- 21.26+/-2.13 years	female-21.27+/- 2.22 years.
Height	male-165.55+/- 7.25 cm	female-157.97+/- 7.95 cm.
Weight	male-63.53+/- 6.67 kg	female- 54.85+/- 5.41 kg.
Body mass index(BMI)	male-23.15+/-1.02	female-21.94+/-0.79
Mean 6MWT distance	male- 570.21+/-35.77 meters	female-494.27+/-34.24 meters.

1.Strong correlation between heaight, weight, BMI and 6MWT observed in both males and females. Table 2.

**Table 2:** 

	Males	Females
Height: P( Predicted)	< 0.0001	< 0.0001
r(coefficient)	0.772	0.862

Mean 6MWT distance by males was higher than that of females for the same height. Table 3.

**Table 3:** 

Weight:P	< 0.0001	< 0.0001
r	0.165	0.125
BMI: P	< 0.0001	< 0.0001
r	0.165	0.125

## Conclusions

Mean 6MWT distance was : male- 570.21+/-35.77 meters, female-494.27+/-34.24 meters

Correlation was strong for height,weight,and BMI with the 6MWT distance. Men had higher mean 6MWT distance than women for the same age,weight and BMI.
